# Functional identification of an antennal lobe DM4 projection neuron of the fruit fly

**DOI:** 10.1186/1471-2202-15-S1-P5

**Published:** 2014-07-21

**Authors:** Aurel A Lazar, Chung-Heng Yeh

**Affiliations:** 1 Department of Electrical Engineering, Columbia University, New York, NY 10027, USA

## 

A rich set of genetic tools and extensive anatomical data make the olfactory system of the fruit fly a neural circuit of choice for studying function in sensory systems. Though a substantial amount of work has been published on the neural coding of olfactory sensory neurons (OSNs) of the fruit fly, yet little is known how projection neurons (PNs) encode time-varying odor stimuli [1]. Here we address this question with *in vivo* experiments coupled with a phenomenological characterization of the spiking activity of PNs.

Recently, a new class of identification algorithms called Channel Identification Machines (CIMs) [2] was proposed for identifying dendritic processing in simple neural circuits using conditional phase response curves (cPRCs) [3]. By combining cPRCs with the reduced project-integrated-and-fire neuron (PIF) model [4], the CIM algorithms identify a complete phenomenological description of spike generation of a biological neuron for weak to moderately strong stimuli. Moreover, the identification method employed does not require white noise stimuli nor an infinitesimal pulse injection protocol as widely used in the past [5].

**Figure 1:**
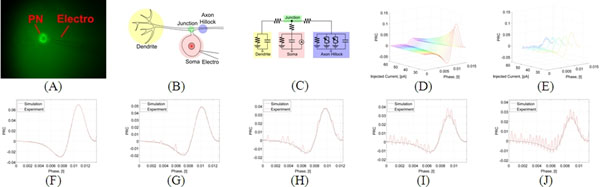
**(A)** PN with GFP expression under 60x magnification; **(B)** Schematic of the anatomy of the PN; **(C)** Circuit diagram of the simulated PN model; **(D)** Identified PRC of the simulated PN model; The injected current varies from 25 [pA] to 60 [pA] with step size 1 [pA]; **(E)** Identified PRC of PN. The injected current varies from 30 [pA] to 50 [pA] with step size 5 [pA]; **(F-J)** Comparison between identified PRC of simulated PN model and the *in vivo* PN; Injected current value is 30 [pA] to 50 [pA] with step size 5 [pA] from (F) to (J). Time is in seconds.

Here we identify the PNs both *in silico* and *in vivo*. Starting with simulations, we investigate the feasibility of the CIM method on PNs modeled as pseudo uni-polar neurons *in silico*, as shown in Figures 1.(B) and 1.(C). We then systematically convert the CIM method into a step-by-step experimental protocol, and carry it out *in vivo* by injecting currents into PNs using the patch clamping technique [6,7]. A snapshot of PN patching is depicted in Figure 1.(A).

We demonstrate that the CIM method accurately identifies the cPRCs of the *in silico* PN model for a wide range of bias currents, as shown in Figure 1.(D). Moreover, the new method also accurately identifies a set of cPRCs of PNs *in vivo*, as shown in Figure 1.(E). For comparison, we tune the identified cPRCs of the *in silico* PN model to fit the *in vivo* identified cPRCs of biological PNs. We demonstrate that: (i) the new method accurately identifies the cPRCs of PNs for small bias currents; (ii) the accuracy of the cPRC is qualitatively lower during the refractory period, as depicted in Figures 1.(F-J).
